# Continuous-Wave Coherent Raman Spectroscopy via Plasmonic Enhancement

**DOI:** 10.1038/s41598-019-48573-8

**Published:** 2019-08-20

**Authors:** Yashar E. Monfared, Travis M. Shaffer, Sanjiv S. Gambhir, Kevin C. Hewitt

**Affiliations:** 1 Department of Physics and Atmospheric Science, Dalhousie University, Halifax, NS Canada; 2 Department of Radiology, Molecular Imaging Program at Stanford University (MIPS), Stanford, CA USA; 3 Departments of Bioengineering and Materials Science & Engineering, Bio-X Program, Stanford, CA USA

**Keywords:** Nanophotonics and plasmonics, Nanoparticles

## Abstract

In this paper, we report a successful combination of stimulated Raman spectroscopy (SRS) and surface-enhanced Raman scattering (SERS) using cw laser sources and gold/silica nanoparticles with embedded reporter molecules. We describe the preparation method for our gold/silica nanoparticles as well as the effect of probe wavelength, pump and probe power, polarization and sample concentration on the cwSESRS signal. Altogether, a stable ~12 orders of magnitude enhancement in the stimulated Raman signal is achieved because of the amplification of both pump and probe beams, leading to the detection of pico-molar nanoparticle concentrations, comparable to those of SERS. The coherent Raman spectra matches the incoherent conventional Raman spectra of the reporter molecules. Unlike conventional incoherent SERS this approach generates a coherent stimulated signal of microwatt intensities, opening the field to applications requiring a coherent beam, such as Molecular Holography.

## Introduction

Raman spectroscopy is an inelastic light scattering technique which yields a “spectral” barcode of the compound under study^1^. The use of novel Raman spectroscopy techniques in different areas of clinical research such as early cancer detection^2^, treatment response studies^3^, and new therapy development^4^ is steadily growing. For example, Raman spectroscopy is a powerful tool for studying molecular distributions in tissue samples^5^, and for imaging *in vivo* brain tumors in mice^6^ and humans^7^. However, the applicability of conventional Raman spectroscopy is limited by the inherently weak Raman scattering cross section of molecules compared with other techniques like fluorescence spectroscopy^8^. This weak signal can make detecting low-density samples (on the order of low nanomolar or picomolar) almost impossible^9^. To overcome these inherent problems, techniques based on vibrational pumping such as stimulated Raman spectroscopy (SRS)^10,11^ or coherent anti-Stokes Raman spectroscopy (CARS)^12,13^ can be utilized. In SRS, the interaction of two light beams (Stokes and pump) whose frequency difference matches a molecular vibration frequency generates energy gain at the Stokes frequency at the expense of the pump^14,15^. Furthermore, in contrast to incoherent scattering in conventional Raman spectroscopy, SRS can provide a coherent beam which can have application in, for example, holography.

Currently, most of the SRS experiments employ a variety of ultra-fast pulsed laser sources^16,17^. There are several inherent problems associated with the use of ultra-short laser pulses such as their cost and more complex optical components which can be a major drawback for future commercialization^18,19^. In addition, the high peak intensity of pulses can be potentially harmful to living cells due to the photo-damage effect^19–21^. To solve these problems, one can use continuous wave (cw) laser sources instead as a cost-effective solution. The major drawback is the fact that the cwSRS signal is nearly three orders of magnitude weaker than SRS using ultra-fast laser sources^19^. Therefore, it is still impossible to detect species at trace concentrations (nM - pM) using low-power cw laser sources.

Luckily, there is a solution for further signal enhancement using metal nanoparticles^22^. The discovery of a Raman signal enhancement triggered by a nearby metal surface has resulted in important developments in spectroscopy and medical imaging. For example, Davis *et al*.^23^ recently demonstrated the application of nanoparticles for the detection of bladder cancer using surface-enhanced Raman spectroscopy (SERS). Recently, Momenpour *et al*.^24^ studied the application of SERS for detection of polycystic ovary syndrome in patients, and Alattar *et al*.^25^ demonstrated a method for differentiation between hematopoietic stem cells using SERS. Although the effect of plasmonic-assisted signal enhancement is well known, the mechanism of enhancement, and the effects of laser sources and sample characteristics on SERS signal are difficult to explain using a simplified theory. Therefore, a wide range of samples and molecular species are used to study the fundamentals of such signal enhancements using theoretical^26^ or experimental^27,28^ approaches.

The combination of surface-enhancement and SRS to detect a gain in the Raman signal is an attractive area of research^27^. For instance, Frontiera *et al*.^28^ reported surface-enhanced stimulated Raman spectroscopy (SESRS) with femtosecond pulsed lasers using gold/silica nanoparticles. Previous studies on pulsed-SESRS show that one can detect a Raman signal for a sample of pico-molar concentration using nanoparticles enhancement and a power density of 0.01 MW cm^*−*2^^28,29^. These values are three to four orders of magnitude lower than that required for conventional SRS. Furthermore, as conventional SRS samples are usually in the micro-molar range, this adds more than three orders of magnitude to the enhancement. Therefore, one can expect to see a large enhancement in the pulsed SESRS signal compared to conventional SRS experiments^28,29^.

However, the SESRS with cw sources can offer a wider range of Raman spectroscopy applications. The application of low- power cwSRS in neat benzene was demonstrated for the first time by Owyoung in 1977^30,31^. We previously demonstrated the possibility of SESRS using cw laser sources (cwSESRS) and commercially available Oxonica nanoparticles for the first time^28^. It has been demonstrated that the surface enhancement leads to detecting samples using focused pump and Stokes beams with a power of around 100 mW^29^. In this paper, we describe the synthesis of gold/silica nanoparticles with a similar architecture employing embedded reporter molecules. Using these samples, we study the role of laser power, polarization and sample concentration on the cwSESRS signal and provide a description of the plasmonic enhancement process using gold/silica nano-probes. The results clearly demonstrate the ability of cwSESRS to detect picomolar quantities because of the corresponding *∼*10^−12^ orders of magnitude enhancement of the product of pump and Stokes beams.

## Sample Preparation

Gold/silica nanoparticles (NPs) were synthesized using methods previously described in the literature^32,33^. A 1 L solution of 0.25 mM gold chloride trihydrate (HAuCl_4_) was brought to boiling. 7.5 mL of 1%(w/v) sodium citrate tribasic dihydrate was added and heated for 10 minutes, after which the dispersion was cooled to room temperature. The NPs were purified via centrifugation (7000 *rcf*, 10 minutes) followed by dialysis (MWCO 3.5 kDa) overnight.

The gold NP dispersion (1 mL, 0.5 nM) was added to isopropanol (12 mL), followed by either embedded reporter A (ERA) molecule which is BPE trans-1,2-bis(4-pyridyl)-ethylene or embedded reporter B (ERB) molecule which is 1,2-di(4-pyridyl) acetylene molecule (ERB, 50 *µ*L, 50 mM), in dimethyl-sulfoxide (DMSO). After 15 seconds, 50 *µ*L of 99.99% tetraethyl orthosilicate (TEOS) and 300 *µ*L 28% (v/v) ammonium hydroxide was added. After 20 minutes, the as-synthesized SESRS-NPs were centrifuged (7000 rcf, 5 minutes) followed by resuspension in ethanol.

**Figure 1:**
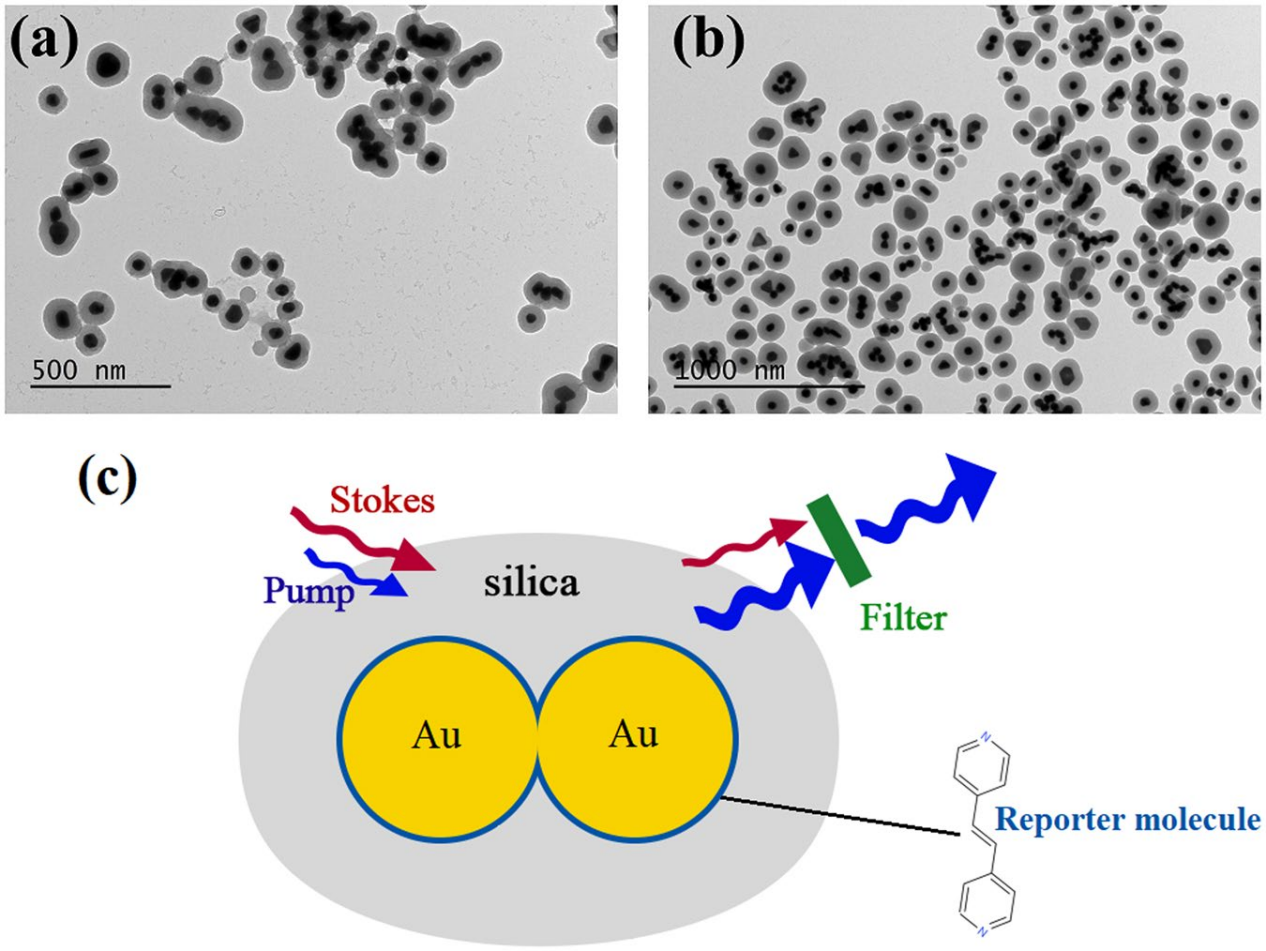
TEM image of particles in samples: (**a**) ERA (embedded reporter A) and (**b**) ERB (embedded reporter B). Note that the dark spots in TEM image are gold NPs and the lighter color around the gold NPs are silica shells. (**c**) Schematic depiction of the gold/silica nano-probes used in the cwSESRS experiment.

All reagents are from Sigma Aldrich except 1,2-Bis(4-pyridyl) acetylene which was provided by Synquest laboratories. The transmission electron microscopy (TEM) images of the resulting samples and schematic depiction of nano-probes in cwSESRS experiment are shown in Fig. 1.

To prepare TEM images of the samples, we used a JEOL-JEM1400. As seen in Fig. 1, there are gold NP monomers, dimers, trimers and even tetramers embedded in the silica shell. Interactions between adjacent NPs forming junctions and crevices give rise to a high density of hotspots which contribute to the field enhancement. These high density hotspot feature of the samples in our experiments are similar to the Cabot Corporation nano-probes (previously known as Oxonica) described in^34^. The hydrodynamic diameter was measured using dynamic light scattering (DLS) provided by Nano-ZS. The DLS diameter of ERA and ERB are 132.4 nm 156.8 nm, respectively, while gold NP diameters are approximately 60 nm. Finally, gold NP concentrations were measured with a NS-500 nano-particle tracking analyzer provided by Nanosight. The NP concentration of ERA and ERB is 488 pM and 679 pM, respectively.

## Theory

In SRS, laser light from two different sources interact with each other to generate a Raman signal. When the frequency difference of the two laser beams match a vibrational mode of the interacting molecule, the pump beam experience a loss of intensity which is called stimulated Raman loss (SRL), and the Stokes (probe) experience a gain of intensity which is called stimulated Raman gain (SRG)^28^. Following the same procedure described in^30,31^, the power gain at the Stokes frequency (*P*_*SRG*_) induced by a pump beam (*P*_*p*_) is approximately given by

1 $${P}_{SRG}=\frac{{C}_{1}}{{C}_{2}}{Im}{\chi }_{1111}^{(3)}(-\,{\omega }_{p},{\omega }_{s},{\omega }_{p},-\,{\omega }_{s}){P}_{s}{P}_{p},$$

where *P*_*s*_ is the Stokes power, *P*_*p*_ is the pump power and *Im*$Im{\chi }_{1111}^{(3)}(-\,{\omega }_{p},{\omega }_{s},{\omega }_{p},-\,{\omega }_{s}){P}_{s}{P}_{p},$ is the imaginary part of the third-order nonlinear susceptibility. C_1_ is given as

2 $${C}_{1}=\frac{384{\pi }^{4}}{cn}$$

where *c* is the speed of light in vacuum and *n* is the refractive index of sample at the Stokes wavelength. *C*_2_ is given as

3 $${C}_{2}=\frac{{(2\pi c)}^{2}}{{\omega }_{p}{\omega }_{s}}$$

The sample characteristics, including Raman cross section of the molecules and sample concentration, manifest themselves in the imaginary part of the third-order nonlinear susceptibility of the sample. can be written as^30,31^

4 $${Im}{\chi }_{1111}^{(3)}(-{\omega }_{p},{\omega }_{s},{\omega }_{p},-{\omega }_{s})=\frac{N{c}^{3}}{48\hslash {\omega }_{S}^{4}}{\sigma }_{Raman}L({\omega }_{p}-{\omega }_{s})$$

where σ_Raman_ is the differential peak Raman cross section, *N* is the number of molecules in the illuminated volume, ħ is the reduced Planck’s constant and *L(ω*_*p*_ − *ω*_*s*_) is a lineshape function which is equal to 1 at the Raman resonance frequency and is a function of frequency detuning *(ω*_*p*_ − *ω*_*s*_). As we can see in Eqs (1–4), the magnitude of the SRG is directly related to the product of Stokes and Pump powers, and sample concentration. Unlike CARS where there is a quadratic dependence on powers, the gain relationship in cwSRS is linear with pump and Stokes powers and is independent of focusing effects or phase-matching terms^31^.

The enhancement factor (EF), which is defined as the ratio of SERS signal to normal Raman scattering signal, is usually used to determine the effect of nanoparticles in signal enhancement. SERS enhancement may arise from resonance Raman effects, chemical effects and/or electro-magnetic effects^26^. The electro-magnetic effects are usually the dominant effect in SERS as the oscillating charge causes the re-radiation of the electric field. For Oxonica nano-probes, EF are estimated to be around 3 × 10^7^ with 785 nm excitation^34^. We expect to see similar values (in the same order of magnitude) for our samples as the samples have similar plasmonic enhancement mechanisms.

## cwSESRS Experiment

**Figure 2:**
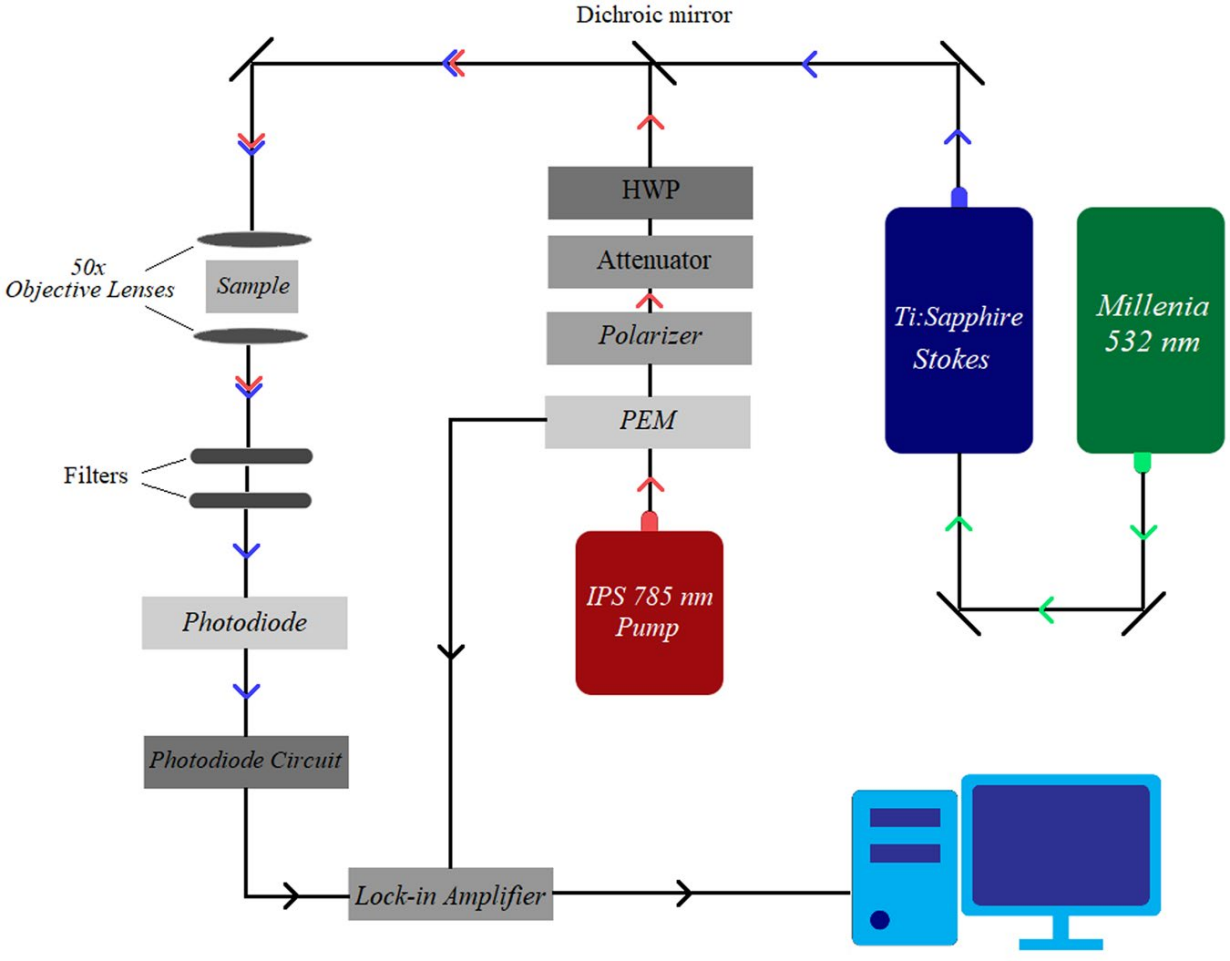
Schematic of the cwSESRS experiment, showing the modulated pump beam and unmodulated Stokes beam paths (PEM is the photo-elastic modulator and HWP is the half-wave plate).

A schematic of the cwSESRS experiment is shown in Fig. 2.

Two different CW laser sources are needed and at least one of them should be tunable over a range of wavelengths. We use an IPS 785 nm (90 mW) cw laser source as the pump beam, and the output of a Spectra Physics 3900-s Ti:Sapphire laser cavity, pumped by a Millennia-Xs 532 nm (10.5 W) laser, as a tunable Stokes beam (861–871 nm). To modulate the pump beam, a Hinds photo-elastic modulator (PEM) 90 with an IFS-50 fused silica head modulates the polarization of the laser beam at 50 kHz. The PEM operates in a quarter-wave retardation regime. A Melles Griot 03-PTH005 linear prism polarizer then passes only vertically polarized light and therefore, the pump beam is modulated at a frequency of 100 kHz. We use an optical attenuator and half-wave plate to control the pump power and polarization state in the experiment. We then align the pump and Stokes beams using a Semrock dichroic mirror (LPD02-785RU). The conjugated beams are then focused onto the sample using a long working distance (50x) objective lens. A 30 *µ*m pinhole is placed in front of the 50x lens, and a 2 *µ*m pinhole is placed in the sample position to ensure both beams are aligned. The pinholes are removed, and the sample positioned where the 2 *µ*m pinhole was originally found. A second 50x objective lens is then used to collect and collimate the transmitted beam.

To measure the light intensity, a Thorlabs SM1PDA1 silicon photodiode is used. The photodiode responsivity at the excitation wavelengths are: 0.646 *AW*^−1^ at 870 nm, 0.655 *AW*^*−*1^ at 880 nm and 0.563 *AW*^*−*1^ at 785 nm. Since the SRG signal is expected to be small, a lock-in amplifier is used to demodulate the SRG signal from the electronic and laser noise. We use a Stanford Research Instrument SR510 lock-in amplifier which offers fast phase control as well as computer control. The 100 kHz reference square wave from the PEM is used for the reference in the lock-in amplifier. We set the pre-time constant of the lock-in amplifier to one second. The SRG signal is extracted from the output Stokes beam by performing a phase scan of the phase difference between the Stokes and reference wave, with an accumulation time of about 60 seconds.

To measure SRG, we block the pump beam by placing two Semrock optical long-pass filters (LP02-785RU and BLP01- 785R) in the beam path (same direction as the incident beam), allowing only the Stokes beam to pass to the detector and prevent optical leakage from the pump beam. We confirmed that no pump beam leaks to the detector by monitoring the output in the absence of the Stokes beam. Also, the unmodulated Stokes beam does not create a signal in the absence of the pump beam. Only when the unmodulated Stokes beam signal is altered by the modulated pump in the sample, a signal at the lock-in frequency observed.

Finally, to generate conventional Raman spectra, we used an InVia Raman microscope (Renishaw) with a 785 nm diode laser excitation and CCD detector. Raman spectra were collected from a solution of ERA (0.244 nM) and ERB (0.340 nM) through a 5x objective. Laser output at the microscope objective was determined to be 20 mW.

## Results and Discussions

### Raman spectra

**Figure 3:**
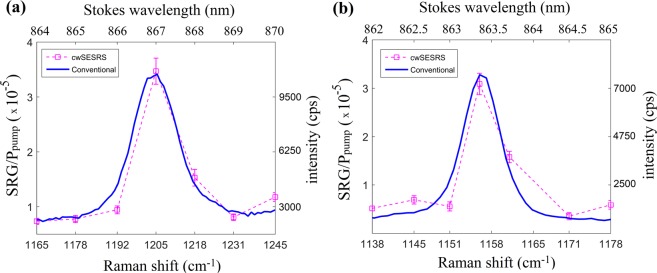
Conventional incoherent Raman spectra (solid blue) using a 785 nm excitation, and SRG spectra using cwSESRS (dotted magenta) for (**a**) ERA (embedded reporter A) and (**b**) ERB (embedded reporter B). Note that the pump was fixed at 785 nm with a power of 34 mW and Stokes beam was used to scan a wavelength range in vicinity of the sample prominent mode.

Figure 3 shows the cwSESRS spectra of our samples (ERA and ERB) using a pump power of 34 mW (785 nm) and Stokes power of 663 mW. The sample is in aqueous solution, sandwiched between glass cover slips which are separated by 2 mm using an o-ring.

Note that the pump wavelength is fixed at 785 nm during the experiments and the Stokes is swept across a range of wavelengths near a Raman vibrational mode of the reporter. The experiment is repeated three times to find the uncertainty and error bars. As seen in Fig. 3(a), the signal generated from ERA match the conventional Raman spectra, as expected for the SRS approach. For ERA which has trans-1,2-bis(4-pyridyl)-ethylene (BPE) as the Raman reporter molecule, the 1205 *cm*^*−*1^ normal mode is the most prominent in our cwSESRS spectra, consistent with BPE spectra in the previous work^29^. This normal mode corresponds to the symmetric stretching of the C-C vibrations and bending vibrations of pyridyl ring C-N bond^35^. In Fig. 3(b), one can readily observe the agreement between the cwSESRS spectra and conventional incoherent Raman spectra for ERB (embedded reporter B) near 1160 *cm*^*−*1^. It should also be noted that the limit of detection for ERA and ERB was found to be 200 × 10^−14^ M and 500 × 10^−13^ M, respectively, using a 785 nm laser (38.9 mW) and 10× objective.

### Effect of source power

**Figure 4:**
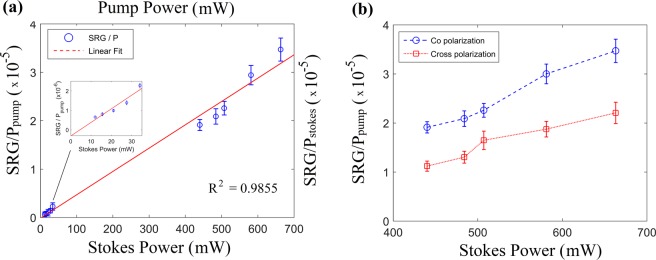
(**a**) The relationship between pump and Stokes power and measured normalized SRG signal in ERA (embedded reporter A). The NP concentration is 0.488 nM, and Stokes and pump wavelengths are fixed at 867 nm and 785 nm, respectively. The inset show the relationship between normalized SRG signal and Stokes power where pump power is 12–34 mW. (**b**) The effect of polarization of the laser sources on the SRG signal in the experiment using ERA. The Stokes wavelength is fixed at 867 nm, concentration of NPs is 0.488 nM, and the pump (785 nm) power is 34 mW.

The effects of pump power and Stokes power on the stimulated Raman gain signal was investigated as shown in Fig. 4(a). The pump power was varied between 12–34 mW using an optical attenuator, while the Stokes power was varied between 440–663 mW by varying the 532 m pump source. According to Eq. (1), one expects a linear relationship between pump and Stokes powers and the SRG signal. As clearly see in Fig. 4(a), the relationship between pump power, Stokes power and normalized SRG signal (normalized to the source power) is linear (χ = 0.9855).

From this experiment, we can estimate the imaginary part of third order nonlinear susceptibility of the sample using Eqs (1–4) to be 14 × 10^*−*13 ^cm^3^/erg for the 1205 cm^*−*1^ normal mode, which is more than eight times larger than that of the corresponding normal mode found at 992 cm^*−*1^ (ring breathing vibration) in neat benzene (1.59 × 10^*−*13 ^cm^3^/erg), described in Owyoung’s experiments^30,31^. Note that the differential Raman cross-section of benzene molecule is approximately 4 × 10^*−*30 ^cm^2^/sr^36^. Considering the differential Raman cross-section of 21 × 10^*−*30 ^cm^2^/sr^37^ for BPE and pico-molar concentration of particles in the sample, the pump and Stokes pulses must contribute a factor of 10^10^–10^12^. This is consistent with an EF of 10^7^ for 785 nm excitation and 10^5^–10^6^ for the 867 nm Stokes beam^34^, as suggested theoretically by Chng *et al*.^27^.

The linearity exhibited in Fig. 4(a) also suggests no sample damage as the signal was reproducible during the experiments; unlike previous work using pulsed lasers which exhibit an unsatisfactory saturation of the SRG indicative of damage^28^.

### Effect of source polarization

As shown in Fig. 4(b), the SRG signal in co-polarized sources are a lot stronger than that of cross-polarized sources. We suspect that the variation of SRG for cross-polarized light is due to slight depolarization of light inside the nanoparticle solution. The depolarization ratio can be higher than 0.75 during the interaction of light beams with NPs^38,39^.

### Effect of sample concentration

**Figure 5:**
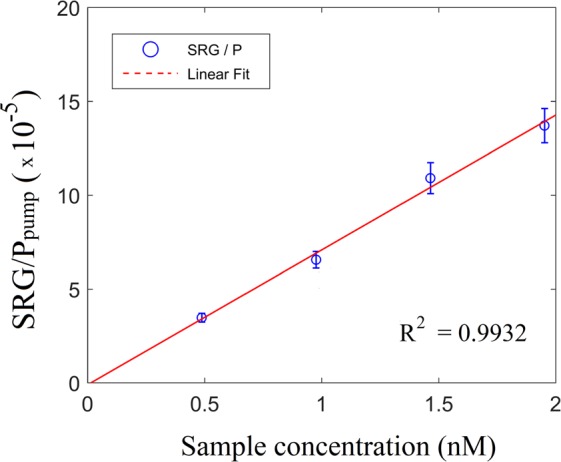
cwSESRS signal dependence on the sample concentration in ERA (embedded reporter A). The Stokes wavelength (867 nm) and the pump (785 nm) powers are 663 mW and 34 mW, respectively.

The effect of sample concentration on the SRG signal is shown in Fig. 5. To alter the concentration, we used an Eppendorf 5810R refrigerated centrifuge to spin the nano-particle solution at 3000 rcf for 30 minutes. After removing the supernatant, the sample were suspended in a smaller volume to achieve a range of concentrations: 0.488, 0.976, 1.464 and 1.952 nM. As seen in Fig. 5, the normalized SRG signal is linear (χ = 0.9932) with the concentration of the nano-probes, as expected from Eq. (4).

## Conclusions

Gold/silica nanoparticles with embedded reporter molecules were synthesized by starting with 60 nm gold nanoparticles which are then aggregated (or not) using a Raman reporter molecule trans-1,2-bis(4-pyridyl)-ethylene (BPE) or 1,2-di(4-pyridyl) acetylene (DPA) into monomers, dimers, trimers etc, then embedded in a silica shell. We studied the effects of probe wavelength, pump and probe/Stokes power, polarization and sample concentration on the stimulated Raman signal. With the pump wavelength fixed at 785 nm (34 mW), a tunable Stokes beam (~663 mW) is swept across a range of wavelengths near a vibrational mode of the Raman reporter. The signal generated from BPE and DPA match the conventional Raman spectra near 1200 cm^−1^ or 1160 cm^−1^, respectively, as expected for stimulated Raman scattering.

Our studies revealed that the stimulated Raman gain signal varies linearly over the sample concentration (0.488–1.952 nM; χ = 0.9932)) and pump and probe powers (12–34 mW and 440–663 mW, respectively; χ = 0.9855)) investigated. There is a reduction in the stimulated signal using crossed-polarized sources which may be due to depolarization effects. Altogether the ~12 orders of magnitude surface enhancement of the combined pump and probe beams in cwSESRS allows one to detect samples with low nano-molar and pico-molar concentrations - levels similar to those of SERS despite the fact that SRS is a third-order effect. The most significant advantage of this approach is beam coherence, and the low cost of cw sources. Unlike conventional incoherent SERS, cwSESRS produces a coherent stimulated emitted beam (of a few nanowatts) which opens up a wide array of applications requiring coherent beams, such as holography.

## Data Availability

Data generated or analysed during this study are included in this published article (Raman spectra, concentration of NPs, SRG signal etc).
